# Nasal Immunization Confers High Avidity Neutralizing Antibody Response and Immunity to Primary and Recurrent Genital Herpes in Guinea Pigs

**DOI:** 10.3389/fimmu.2016.00640

**Published:** 2016-12-26

**Authors:** Josefine Persson, Yuan Zhang, Thorunn A. Olafsdottir, Karolina Thörn, Tina M. Cairns, Frank Wegmann, Quentin J. Sattentau, Roselyn J. Eisenberg, Gary H. Cohen, Ali M. Harandi

**Affiliations:** ^1^Department of Microbiology and Immunology, Institute of Biomedicine, Sahlgrenska Academy, University of Gothenburg, Gothenburg, Sweden; ^2^Department of Microbiology, School of Dental Medicine, University of Pennsylvania, Philadelphia, PA, USA; ^3^Sir William Dunn School of Pathology, University of Oxford, Oxford, UK; ^4^Department of Pathobiology, School of Veterinary Medicine, University of Pennsylvania, Philadelphia, PA, USA

**Keywords:** genital herpes, nasal immunization, neutralizing antibody, competitive surface plasmon resonance assay, sexually transmitted infection, vaccine

## Abstract

Genital herpes is one of the most prevalent sexually transmitted infections in both the developing and developed world. Following infection, individuals experience life-long latency associated with sporadic ulcerative outbreaks. Despite many efforts, no vaccine has yet been licensed for human use. Herein, we demonstrated that nasal immunization with an adjuvanted HSV-2 gD envelope protein mounts significant protection to primary infection as well as the establishment of latency and recurrent genital herpes in guinea pigs. Nasal immunization was shown to elicit specific T cell proliferative and IFN-γ responses as well as systemic and vaginal gD-specific IgG antibody (Ab) responses. Furthermore, systemic IgG Abs displayed potent HSV-2 neutralizing properties and high avidity. By employing a competitive surface plasmon resonance (SPR) analysis combined with a battery of known gD-specific neutralizing monoclonal Abs (MAbs), we showed that nasal immunization generated IgG Abs directed to two major discontinuous neutralizing epitopes of gD. These results highlight the potential of nasal immunization with an adjuvanted HSV-2 envelope protein for induction of protective immunity to primary and recurrent genital herpes.

## Introduction

HSV-2 is the leading cause of genital herpes, one of the most prevalent sexually transmitted infections (1). HSV-2 enters *via* the epithelium of the genital tract mucosa, where it replicates, and transfers to the sensory neurons innervating the infected area. The virus establishes life-long latency in the dorsal root ganglia (DRG), where it can sporadically reactivate and cause recurrent genital herpes disease (2). Re-infection of epithelial cells during recurrent outbreaks may lead to symptomatic or asymptomatic HSV-2 shedding (3). During the silent latent phase of HSV infection the latency-associated transcript (LAT) is the only RNA transcribed and is therefore used as a latency marker (4).

To date, no vaccine against genital herpes is available for human use. A few subunit vaccine candidates using systemic immunization have reached advanced clinical trials with disappointing results (5–7). Earlier studies by our group and others have demonstrated that nasal immunization with adjuvanted recombinant HSV-2 glycoproteins can confer protective immunity to primary genital herpes infection in mice (8–12). However, little is known about the potential of mucosal immunization for induction of protective immunity to primary HSV-2 infection, establishment of latency and recurrent genital outbreaks in guinea pigs, which is the most clinically relevant animal model of the disease [reviewed in Ref. (13)]. Likewise, while the importance of antibodies (Abs) in protective immunity to primary genital herpes has been extensively studied in mice (14–19), the neutralizing characteristics of the Ab response elicited after mucosal immunization remains poorly understood. In the present study, we attempted to investigate these important issues in a guinea pig model of the disease. We opted to use gD protein as a prototype HSV-2 antigen owing to its essential role in mediating the binding of the virus to two major cellular receptors (herpesvirus entry mediator (HVEM) and nectin-1) and activating the downstream components of the viral fusion machinery i.e., gH/gL and gB (20). Furthermore, gD possesses the ability to induce virus-neutralizing Abs, and as such its antigenic structure has been well studied using a large panel of anti-gD monoclonal Abs (MAbs) (21, 22). We employed a biosensor-based MAb-blocking assay along with HSV-2 neutralization assay to decipher whether nasal immunization with adjuvanted gD protein could elicit high avidity IgG Ab responses against epitopes overlapping those of well-characterized MAbs and whether this IgG Ab response has neutralizing properties.

We report herein that nasal immunization with CpG-adjuvanted gD protein evokes high avidity neutralizing IgG Abs against two discontinuous gD epitopes overlapping those of well-characterized MAbs. Furthermore, we showed that nasal immunization confers protective immunity to primary genital herpes, establishment of latency, and recurrent outbreaks in guinea pigs. These results provide new insights into the potential of nasal immunization to elicit protective immunity to genital herpes.

## Materials and Methods

### Animals

Female Dunkin-Hartley guinea pigs (350–450 g) and C57BL/6 mice (7–9 weeks old) were purchased from Charles River, Germany. Female μMT mice (8–10 weeks old) on C57BL/6 background were bred in-house (kindly provided by MIVAC, University of Gothenburg, Sweden). The animals were housed under specific-pathogen-free conditions at the Experimental Biomedicine Animal Facility, University of Gothenburg. When required, the animals were sedated with Isofluran (Baxter Healthcare). The guinea pigs were euthanized with an intraperitoneal overdose of pentobarbital, followed by heart removal. The mice were sacrificed by cervical dislocation. All experiments were conducted with the approval (311-12) of the Ethical Committee for Animal Experimentation in Gothenburg, Sweden.

### Virus

HSV-2 strain 333 was grown and titrated in African green monkey kidney cells [GMK-AH1; (23)], according to a previous publication (10).

### Immunization Scheme

Groups of guinea pigs (in total *n* = 15–18) were nasally immunized three times, at 14-day intervals, with 50 µg recombinant HSV-2 gD (24) alone or together with 150 µg CpG ODN 7909 (TCG TCG TTT TGT CGT TTT GTC GTT) (Eurofins MWG/Operon), in 250 µl. Groups of mice (in total *n* = 5) were nasally immunized three times, at 7-day intervals, with 5 µg gD together with 15 µg CpG ODN 1826 (TCC ATG ACG TTC CTG ACG TT) (Eurofins MWG/Operon), in 20 µl. Phosphate buffered solution (PBS) was used for dilutions and a group given PBS was included as negative control in all experiments. The inoculum was divided equally between the nostrils with a micropipette in sedated animals. Three studies were performed for guinea pigs and two studies for the mice. Results shown are pooled from the experiments unless stated otherwise.

### Proliferative Analysis in Guinea Pigs

Four weeks past, the final immunization blood samples were withdrawn from the heart, into a syringe containing heparin (500 U), of guinea pigs under deep sedation. After euthanasia, spleen and para-aortic/iliac lymph nodes (herein referred to as genital lymph nodes (gLN)) were excised. Lymphoid organs were mashed against filter papers and cells pelleted by centrifugation at 400 × *g* (Thermo Multifuge 3S-R), 5 min, 4°C. Erythrocytes in splenocyte suspensions were lysed with ammonium chloride (5 ml/spleen) at 37°C. PBMCs were isolated from blood by using Histopaque 1083 (Sigma-Aldrich). All cells were suspended in Iscove’s medium, containing 10% fetal bovine serum, 100 µg/ml gentamicin sulfate, 50 µM 2-β mercaptoethanol, all from Sigma-Aldrich, in addition to 2 mM l-glutamine (Biochrom). Cells were seeded in sterile 96-well plates (Nunc), 2 × 10^5^ per well, with gD (2 µg/ml) or Concanavalin A (2.5 µg/ml) (Sigma-Aldrich), as positive control, and incubated at 37°C, 5% CO_2_. Selected plates were used to collect cells at 24 and 48 h. The remaining cells were incubated with 1 μCi of [6-^3^H] thymidine (Amersham Biosciences) for 6–8 h, at 72 h, before frozen at −20°C. The cell proliferation was analyzed by a scintillation counter (1450 MicroBeta Trilux) as counts per minute.

Serum samples from guinea pigs. Blood samples were retrieved, by puncturing hind leg *v. saphena*, 4 weeks after the final immunization. Samples were left to coagulate at room temperature (RT), centrifuged twice at 600 × *g* for 5 min at 4°C (Thermo Sci., Fresco 17), and the sera were frozen at −20°C.

### Vaginal Swab Samples from Guinea Pigs

The vaginal vault, of sedated guinea pigs, was swabbed with a 100 mm swab (MicroRheologics) dipped in PBS 4 weeks post-immunization. The brush part was then placed in 300 µl PBS on ice. Supernatants were recovered from vortexed and centrifuged (600 × *g*, 5 min, 4°C) samples and stored at −20°C.

### Analysis of gD-Specific Ab Levels

The content of gD-specific Abs were determined in serum and vaginal swab samples by ELISA, as previously described (10). Horseradish peroxidase-conjugated goat α-guinea pig IgA (Immunology Consultants Laboratory) or IgG (Southern Biotech) antibodies were used for detection.

### Serum IgG Purification

Pearl IgG spin columns (GBiosciences) were used to purify IgG Abs from guinea pig sera according to the manufacturer’s protocol. Briefly, 200 µl resin was added to a column, after which 10-fold diluted serum samples were added in a 500 µl-volume. After purification, samples were adjusted to 0.2 mg/ml based on Nanodrop spectrophotometer (Thermo Scientific) protein concentration. Purified samples were stored at −20°C.

### Avidity Measurements of IgG with ELISA

Avidity of gD-specific IgG Abs in sera was measured using the aforementioned ELISA protocol, with the addition of a potassium thiocyanate (KSCN) elution step (25). Briefly, 100 µl of serum samples, diluted 50-fold in PBS-Tween 20 with BSA (1%), were added to gD-coated plates and incubated for 2 h at 37°C. After washing with PBS-Tween 20, twofold dilutions of KSCN, ranging from 0.125 M to 8 M, or PBS-Tween 20 (representing 100% binding) were added and incubated for 15 min at RT. The reaction was developed and absorbance read at 450 nm. The results were expressed as an avidity index, defined as the M KSCN needed to dissociate 50% of bound IgG Abs.

### Avidity Measurements with Surface Plasmon Resonance (SPR)

Anti-His Abs (His capture kit, GE) were immobilized onto a CM5 sensor chip (~13,800 response unit (RU)), and His-tagged gD was flowed over to immobilize gD at approximately 1,200 RU using a Biacore 4000 SPR instrument (GE Healthcare, Biacore Life Sciences). Purified IgG samples flowed over the chip to observe the binding and dissociation phases. A control flow cell was immobilized with an equimolar amount of BSA, and all data were normalized to sensograms from this control flow cell. All samples were run in two independent experiments with reversed sample order, each consisting of one anti-His flow cell and one control (BSA) flow cell. Relative avidity was determined by taking the average maximal binding during the association phase divided by the dissociation rate constant (kD) to yield the avidity score (RU × s). The results from the two independent experiments were averaged.

### gD-Specific Competitive SPR

Competitive SPR experiments were performed using the dual channel SPR SR7500DC System (Reichert Technologies Life Sciences, Buffalo, NY, USA) with a NiCH 1000 M sensor chip (XanTec bioanalytics GmbH, Düsseldorf, Germany) at RT. Filtered and degassed HBS-EP buffer (10 mM HEPES, pH 7.4, 150 mM NaCl, 3 mM EDTA, 0.005% Tween 20) was used as running buffer. The sensor chip was activated by a 3 min-injection of nickel sulfate solution (40 mM), followed by injection of ethyl dimethylaminopropyl carbodiimide (40 mg/ml) together with N hydroxysuccinimide (10 mg/ml) to the left channel. gD protein (50 µg/ml) was injected to the left channel for 5 min and 1,300 RU was immobilized onto the sensor chip. For surface deactivation, ethanaolamine (1 M, pH 8.5) was injected for 8 min into the left channel, followed by injection of EDTA (350 mM) for 3 min to rinse non-covalently bound protein. The competitive SPR experiment was performed as explained elsewhere (26). Briefly, purified guinea pig serum IgG Abs (100 µg/ml) were injected for 180 s to both the left and the right channel, after which gD-specific mouse MAbs 1D3, DL11, MC2, MC5, MC14, MC23 (22, 27, 28) (75–150 µg/ml) were injected to both channels for 180 s. The right channel was used as a reference, to record background binding. After each round, the surface of the sensor chip was regenerated by injection of NaOH (50 mM) for 1 min until the RU signal returned to the baseline. All injections were performed at a flow rate of 25 µl/min. The blocking activity of each guinea pig IgG sample was calculated for each MAb as a percentage using the following formula: [1 − (RU MAb binding to human IgG-coated chip/RU MAb binding to control chip)] × 100.

### HSV-2 Neutralization Assay

Serum samples, serially diluted in Iscove’s complete medium, were pre-incubated with 300 plaque-forming units (PFU) of HSV-2 strain 333 for 2 h, 37°C, 5% CO_2_. The pre-incubated virus was applied to GMK-AH1 cells, grown 2 × 10^4^ cells/well in sterile 96-well plates (Nunc). The cell plates were left at RT for 1 h, followed by 3 days of incubation at 37°C, 5% CO_2_. Cells were stained with Crystal violet (Sigma-Aldrich), and the cytopathic effect was examined by microscopy in a blinded fashion. HSV-2 neutralization was considered as the highest possible dilution of sera at which a 50% reduction of plaques was observed relative to the virus control.

### Vaginal HSV-2 Challenge

HSV-2 challenge of guinea pigs was conducted 4 weeks post-immunization. The vaginal vault was pre-swabbed with HBSS and 2 × 10^5^ PFU of HSV-2 strain 333 was administered with a micropipette under anesthesia. Three weeks after the final immunization, mice were injected subcutaneously with 3 mg of Depo-Provera (Pzifer), diluted in PBS, in order to increase susceptibility to infection by inducing diestrus. Six days later, the mice were anesthetized and challenged intravaginally with 9 × 10^4^ PFU of HSV-2 strain 333.

### Vaginal Sampling

Vaginal fluids were collected from guinea pigs and mice 3 days post-infection for viral plaque assay. In one of the guinea pig experiments samples were retrieved twice a week during the latent phase of infection (13 days in total) in order to study viral shedding. The vaginal vault of guinea pigs were swabbed and washed with 300 µl Hank’s balanced salt solution. After centrifugation, 200 × *g*, 5 min, 4°C, supernatants were retrieved in order to avoid excess mucus. Two 40 µl-washes were collected from mice in a total volume of 1 ml HBSS. Samples were stored at −80°C until use.

### HSV-2 Plaque Assay

Vaginal HSV-2 levels post-infection were determined by plaque assay as previously described (10). Guinea pig samples were tested in 10- and 50-fold dilutions, while mouse samples were tested in a 10-fold dilution. Virus levels were recorded as PFU/ml vaginal sample.

### Inflammation and Disease

Guinea pigs were monitored daily for 65 days after HSV-2 challenge. The severity of disease was graded as: 0 = healthy, 1 = swelling and/or genital erythema, 2 = a few small vesicles, 3 = several confluent ulcerated vesicles, 4 = ulcerated lesions, purulent inflammation, incontinence, and 5 = large disseminated lesions, severe weight loss, neurological symptoms. Recurrent symptoms were considered as newly emerged genital vesicles (score 2 and above) arising after day 14 post-challenge. Mice were examined daily for up to 14 days post-challenge. The severity of disease was graded as: 0 = healthy, 1 = genital erythema, 2 = moderate genital inflammation, 3 = severe genital lesions, and 4 = hind limb paralysis. Guinea pigs and mice were sacrificed as soon as they displayed signs of severe illness (scores 5 and 4, respectively).

### Isolation of Nervous Tissues

Guinea pigs surviving the HSV-2 infection were sacrificed between day 65 and 70 post-infection and the lumbosacral DRG were collected. Pooled DRG from naive guinea pigs were used as control in the real-time PCR experiments. Spinal cord and lumbosacral DRG were collected from all sacrificed mice, irrespective of day.

### Quantification of IFN-γ mRNA and HSV-2 mRNA/DNA by Real-Time PCR

Guinea pig gLN cells (24 h), splenocytes and PBMCs (48 h) collected after *in vitro* gD recall stimulation, were washed twice in PBS, pelleted and frozen at −80°C. Guinea pig DRG and mouse DRG/spinal cord were collected in RNAlater and subsequently stored at −80°C. All reagents for RNA preservation, RNA/DNA extractions as well as cDNA synthesis (Qiagen GmbH Germany) were used following the manufacturer’s recommendations. Total RNA was extracted with RNeasy mini kit, co-purification of RNA/DNA was performed with Allprep DNA/RNA mini kit and DNA from vaginal swab samples was purified by DNeasy blood and tissue kit. All RNA/DNA preparations that passed quality tests were used for downstream analysis. The concentration and absorbance ratios of RNA/DNA were determined with a Nanodrop spectrophotometer. The QuantiTect Reverse Transcription kit was used for cDNA preparation, including a DNA wipeout step. Real-time PCR on the cDNA (50 ng/reaction) and DNA (100 ng/reaction) samples was carried out in duplicate. MicroAmp Optical plates and Power SYBR Green PCR Master Mix were used for detection and plates were run in an ABI 7500 real-time PCR system, all three from Applied Biosystems, UK. Primers guinea pig IFN-γ mRNA were forward, 5′- AGGAGACGATTTGGCTCTGA-3′, and reverse, 5′-GAAGTTCTTTGGACCTGATCG-3′ (29). Primers HSV-2 LAT mRNA were forward, 5′-gtcaacacggacacactcttttt-3′, and reverse, 5′-cgaggcctgttggtctttatc-3′ (30). The size of the PCR product for each primer set was verified on ethidium bromide agarose gel. The amplification efficiency of the PCR reactions was verified by analyzing samples in serial dilution. The specificity of primers targeting LAT was confirmed by submitting the PCR product (from two positive samples) for sequencing, after which a Blast search was performed. Housekeeping gene hypoxanthine phosphoribosyl transferase was used for normalization, using primers forward, 5′-AGGTGTTTATCCCTCATGGACTAATT-3′, and reverse, 5′-CCTCCCATCTCCTTCATCACAT-3′. Data were analyzed by the ΔC_t_ method. Fold changes of IFN-γ in gD-stimulated cells were calculated against unstimulated cells from the same individual. Fold changes of IFN-γ in DRG were calculated against data from naïve guinea pigs. Fold changes >4 were considered positive. Quantification of HSV-2 DNA was performed by gB gene amplification with primers forward, 5′-TGCAGTTTACGTATAACCACATACAGC-3′, and reverse 5′-AGCTTGCGGGCCTCGTT-3′ (31). HSV-2 DNA (Advanced Biotechnologies) was used for calculation of the number of viral genome copies. The standard curve was based upon eight different concentrations, which ranged from 10^4^ down to 10 genome copies per reaction. The mean *R*^2^ value for the standard curves was 0.985. The detection threshold was 8 genome copies/reaction.

### Statistical Analysis

GraphPad Prism 6 (GraphPad Software) was used for statistical analysis. Data from three groups were analyzed by one-way ANOVA followed by the Tukey multiple comparison test (CI 95%). Data sets involving two groups were analyzed with a two-tailed unpaired *t* test (95% CI). Survival data were evaluated with the Kaplan–Meier method and the Log-rank test. Correlation was computed by non-parametric Spearman test. Differences were considered statistically significant at *p* values of <0.05 (*), <0.01 (**), and <0.001 (***).

## Results

### Nasal Immunization Provides Significant Protection to Primary Genital Herpes Infection in Guinea Pigs

**Figure 1:**
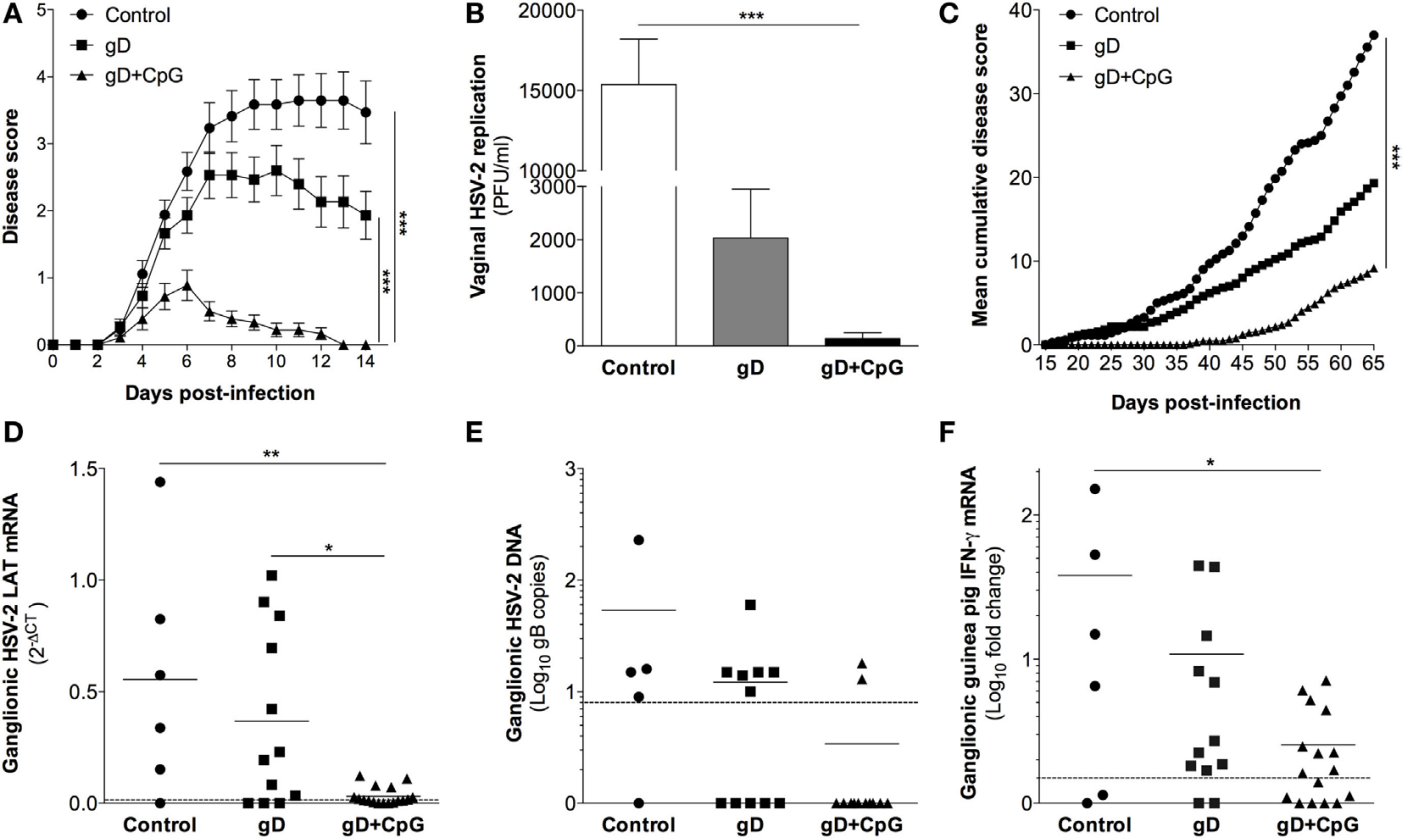
**Nasal immunization in guinea pigs mounts protective immunity to primary genital herpes, establishment of viral latency and recurrent outbreaks**. Groups of female guinea pigs were immunized nasally with gD alone or together with CpG three times with 14 days interval. A control group given the diluent PBS was also included. Four weeks following the last immunization guinea pigs (*n* = 15–18) were challenged vaginally with 2 × 10^5^ plaque forming units (PFU) of HSV-2, thereafter monitored daily for symptoms of disease. The guinea pigs surviving primary infection (*n* = 7–18) were continued to be monitored daily for up to day 65 post-challenge for symptoms of recurrent outbreaks. At day 65–70 post-infection, ganglia were isolated from the animals and RNA was purified alone or co-purified with DNA from the ganglia, and the levels of mRNA and DNA were determined by real-time PCR. **(A)** Disease progression during primary HSV-2 infection (day 0–14 post-challenge). Disease symptoms scored as 0 = healthy, 1 = swelling and/or genital erythema, 2 = a few small vesicles, 3 = several confluent ulcerated vesicles, 4 = ulcerated lesions, purulent inflammation, incontinence, and 5 = large disseminated lesions, severe weight loss, neurological symptoms. Guinea pigs were euthanized as soon as displaying severe signs of illness (score 5). **(B)** The level of vaginal HSV-2 titers on day 3 post-challenge displayed as PFU/ml. **(C)** Disease progression during latent HSV-2 infection (day 15–65 post-challenge) shown as mean cumulative disease score over time (*n* = 7–18). **(D)** RNA extracted from ganglia (*n* = 6–17) was quantified by real-time PCR with primers specific for HSV-2 LAT. The results are shown as 2^−δCT^ of LAT. **(E)** Viral DNA copies in ganglia shown as the number of HSV-2 genome copies per 100 ng DNA (*n* = 5–12). **(F)** IFN-γ transcript in dorsal root ganglia (DRG) (*n* = 6–17) expressed as fold change over the mean expression in naïve guinea pigs. The dotted line in **(D–F)** displays the detection threshold. Results in **(A,B)** are expressed as the mean ± SEM. The end points in **(A,C)** as well as the data in **(B)** and **(D–F)** were analyzed by one-way ANOVA followed by Tukey multiple comparison test (CI 95%), with differences statistically significant at *p* values of <0.05 (*), <0.01 (**), and <0.001 (***).

First, we investigated the potential of nasal immunization for induction of protection against primary genital herpes in a guinea pig model of the disease. Guinea pigs were immunized three times with gD singly or in combination with CpG ODN 7909 or were left unimmunized. Four weeks after the final immunization the animals were inoculated vaginally with 2 × 10^5^ PFU of HSV-2. Following challenge, all unimmunized (17/17) and the majority (12/15) of the gD immunized guinea pigs showed signs of erythema, blisters, and ulcers around the opening of their vaginal vaults and in few animals neurological symptoms emerged (Figure 1A). However, the majority of the gD group survived the infection (12/15), while over half of the controls (10/17) showed severe symptoms requiring euthanizia (Table 1). Among those that recovered from the primary infection, the recovery was faster in the gD immunized animals compared with the unimmunized group (Figure 1A). In sharp contrast, the guinea pigs immunized with gD + CpG displayed no or mild symptoms (7/18) of the disease and all animals survived the primary infection (Figure 1A; Table 1). Furthermore, this group showed virtually no detectable virus titers in their genital secretions (Figure 1B, *p* < 0.001 compared with the control group). In summary, nasal-mucosal immunization with gD + CpG confers strong protective immunity against an otherwise severe primary genital HSV-2 infection in guinea pigs (Table 1).

**Table 1:** **Disease progression in HSV-2 guinea pig model**.

Immunization^a^	Primary infection			Latent infection	
	Skin disease^b^	Replication^c^	Survival^d^	Skin disease^b^	Shedding^e^
Control	15/17	16/17	7/17	7/7	3/13
gD	12/15	11/15	12/15	10/12	7/65
gD + CpG	7/18	5/18	18/18	12/18	7/104

*^a^Guinea pigs immunized according to Figure 1*.

*^b^Individuals displaying minimum score 2 (one or more vesicles)/total individuals challenged*.

*^c^Positive vaginal swabs for HSV-2 replication by plaque assay/total samples tested*.

*^d^Survivors/total individuals challenged*.

*^e^Days tested positive for vaginal HSV-2 gB DNA/total days tested (single experiment)*.

### Nasal Immunization Engenders Protection against Establishment of Latency and Recurrent Genital HSV-2 in Guinea Pigs

After recovery from the primary genital HSV-2 infection, the guinea pigs continued to be monitored daily for 65 days after challenge. In most animals, the first recurrence episode started after day 25 post-challenge (Figure 1C). All controls surviving the primary HSV-2 infection displayed recurrent disease (7/7), with a high episode rate and long-lasting outbreaks (mean recurrent days = 21). In the gD immunized group, the recurrent outbreak was observed in 83% of the survivors, with a relatively high frequency and long-lasting symptoms (mean recurrent days = 13, *ns* compared with the control group). Although several of the gD + CpG group showed mild signs of recurrent disease, the symptoms emerged at a later stage were of significantly shorter duration and less frequent compared with those of the control and the gD immunized guinea pigs (mean recurrent days = 5, *p* < 0.001 compared with the control group) (Figure 1C; Table 1). Importantly, one third of the gD + CpG immunized guinea pigs remained free of symptoms of recurrent genital HSV-2 infection throughout the experiment (Table 1).

Next, we investigated the establishment of latency in the lumbosacral DRG on day 65–70 after challenge in all guinea pigs surviving the primary infection. All but one guinea pig from the control group tested positive for ganglionic LAT mRNA (positive: 5/6, 2^−δCt^ LAT: 0.555 ± 0.214), as did the majority of the gD group (positive: 9/12 positive, 2^−δCt^ LAT: 0.368 ± 0.113) (Figure 1D). In line with the protection data, LAT expression was virtually undetectable in the gD + CpG immunized guinea pigs. Nevertheless, LAT was detectable, albeit at very low levels, in the DRG of the gD + CpG guinea pigs that exhibited recurrent disease (positive: 12/17, 2^−δCt^ LAT: 0.032 ± 0.010).

Next, we quantified HSV-2 DNA copies in the DRG samples by gB-specific PCR. The majority of the DRG samples from the control and the gD group contained HSV-2 DNA (positive: 4/5 control and 6/11 gD), while most gD + CpG immunized guinea pigs tested negative (positive: 2/12) (Figure 1E). The expression of LAT was found to correlate strongly with viral DNA copy number in the ganglia (*r*_S_ = 0.81) (Figures 1D,E; Table 1).

In addition, we assessed IFN-γ mRNA level in DRG as a surrogate marker for latent infection as reported previously (32, 33). Several of the challenged guinea pigs displayed IFN-γ expression in their DRG with a pattern similar to that of HSV-2 LAT mRNA (positive: 4/6 control, 10/12 gD, and 9/16 gD + CpG) (Figure 1F).

Taken together, these results demonstrate that nasal immunization with gD in combination with CpG induced considerable protection against establishment of latency and recurrent genital herpes in guinea pigs.

### Antigen-Specific T Cell and Ab Responses Induced Following Nasal Immunization in Guinea Pigs

**Figure 2:**
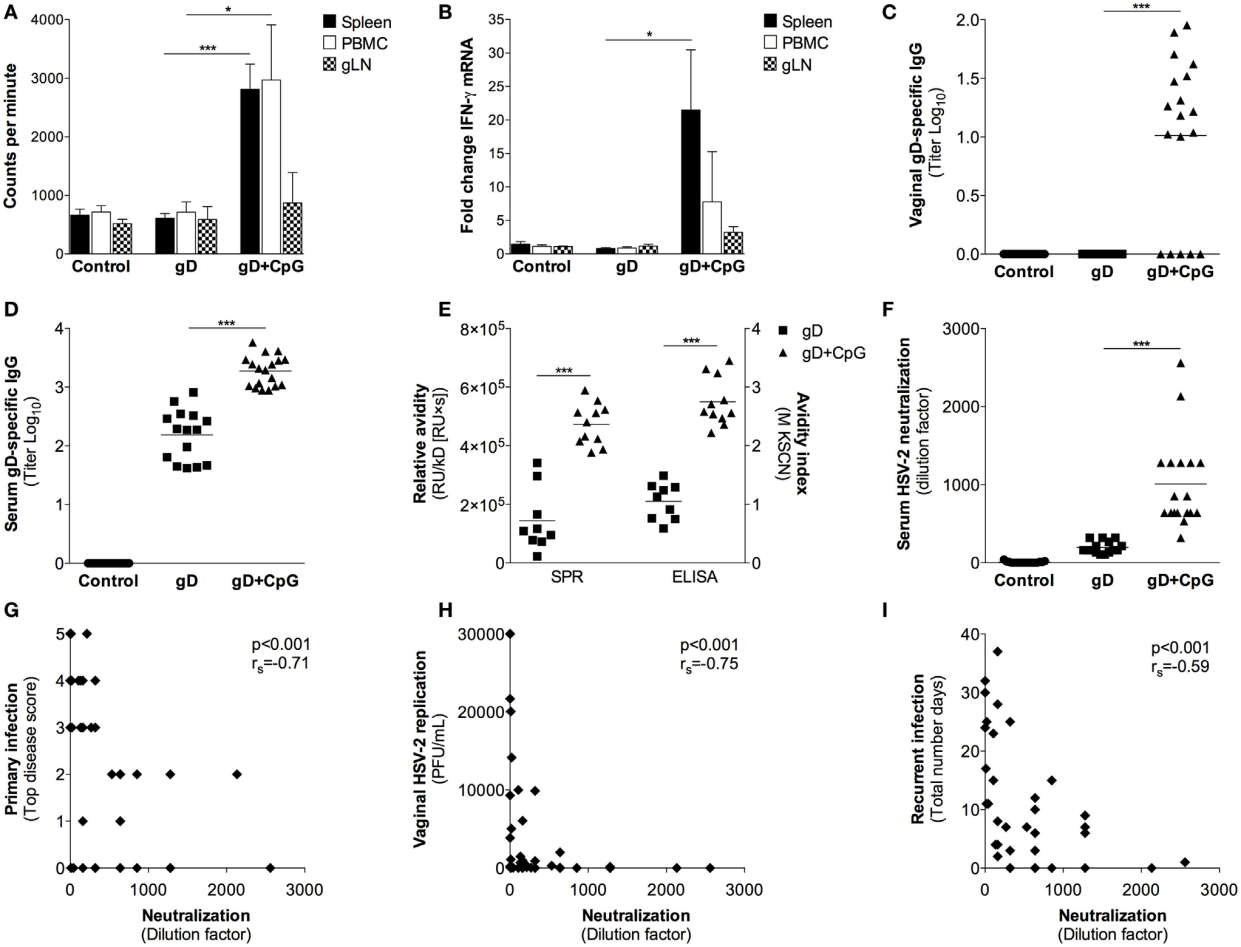
**Characterization of immune responses elicited after nasal immunization in guinea pigs**. Guinea pigs were immunized as explained in Figure 1. Four weeks after the final immunization cells isolated from spleen, blood, and genital lymph nodes (gLN) were re-stimulated with gD protein *in vitro*. **(A)** After 72 h, splenocytes, PBMC and gLN cells (*n* = 4) were harvested and proliferation recorded as counts per minutes. **(B)** The RNA samples obtained from the cultured cells at 24 (gLN) and 48 h (spleen and PBMC) were analyzed for IFN-γ mRNA levels by real-time PCR. Results show IFN-γ expression in gD-stimulated cells expressed as fold change over unstimulated cells. **(C,D)** The content of gD-specific IgG in sera and vaginal swabs taken 4 weeks after the last immunization was determined by a gD-specific ELISA (*n* = 15–18). Results are expressed as titer (Log_10_), based on the mean optical density (OD) value at 490 nm with a threshold of 0.4 above the background OD. **(E)** HSV-2 neutralization effect of sera was examined by a HSV-2 neutralization assay on monolayers of GMK-AH1 cells. The neutralizing properties of sera are presented as the highest dilution factor resulting in 50% reduction of the cytopathic effect. **(F)** Avidity of gD-specific IgG in sera measured by surface plasmon resonance (SPR) and KSCN based ELISA. SPR data presented as avidity score (response unit (RU)/dissociation rate constant (kD)[RU × s]) and ELISA data as avidity index. Serum neutralization capacity plotted against **(G)** primary disease score (top score), **(H)** vaginal HSV-2 replication at day 3 post-challenge and **(I)** the number of recurrent days. The Spearman correlation coefficient is indicated in each plot. Data in **(A,B)** are expressed as the mean + SEM. Individual guinea pig data in **(C–I)** are represented by each dot and the lines in **(C–F)** indicate the mean value for each individual group. Results shown in **(A,B)** are pooled from two independent experiments. Results shown in **(C,F,I)** are pooled from three independent experiments. Data in **(A–E)** were analyzed by a one-way ANOVA followed by Tukey multiple comparison test (CI 95%), while the results in **(F)** were analyzed with a two-tailed unpaired *t* test and 95% CI. Differences were considered statistically significant at *p* values of <0.05 (*), <0.01 (**), and <0.001 (***).

Four weeks after the final immunization, splenocytes, PBMCs, and gLN cells were isolated and tested for proliferative responses and IFN-γ mRNA levels after antigen recall stimulation *in vitro*. The gD immunized guinea pigs did not show any appreciable increase in antigen-specific proliferation and IFN-γ responses over the PBS control group (Figures 2A,B). The gD + CpG group, on the other hand, displayed an antigen-specific proliferative response and IFN-γ expression by their splenocytes, PBMCs and gLN cells (Figures 2A,B).

While the vaginal secretions of the guinea pigs immunized with gD alone contained a modest serum gD-specific IgG Ab response, the guinea pigs given gD + CpG demonstrated significantly higher antigen-specific serum IgG Ab titers in their vaginal secretion samples (*p* < 0.001 compared with the gD group) (Figures 2C,D). No systemic or vaginal gD-specific IgA Abs could be detected in any of the groups (data not shown). Taken together, these data show that nasal immunization could mount systemic antigen-specific T cell responses as well as strong mucosal and systemic IgG Ab responses in guinea pigs.

### Functional Analysis of the Ab Response Induced Following Nasal Immunization in Guinea Pigs

Next, we sought to explore the quality and function of the Ab response induced after nasal immunization in guinea pigs. First, we assessed the avidity of the gD-specific IgG Abs by ELISA and SPR assay. The gD + CpG immunized guinea pigs showed significantly higher avidity of gD-specific IgG Abs compared to those of the gD immunized animals, both by ELISA and SPR (*p* < 0.001) (Figure 2E). To further analyze the function of the vaccine-induced Abs, we analyzed their HSV-2 neutralizing properties. As expected, sera collected from the control group showed no neutralization, and the level was also low for the gD immunized animals (Figure 2E). On the contrary, sera from the gD + CpG group showed significantly more potent HSV-2 neutralization compared with that of the gD group (*p* < 0.001) (Figure 2F). The HSV-2 neutralization effect was shown to be independent of the complement system, as heat-inactivation did not affect the neutralizing properties of the sera (data not shown). There was a significant inverse correlation between serum neutralization capacity and the severity of the primary disease (*r*_S_ = −0.71, *p* < 0.001, Figure 2G), vaginal HSV-2 titers post-infection (*r*_S_ = −0.75, *p* < 0.001, Figure 2H) and number of days with recurrence (*r*_S_ = −0.59, *p* < 0.001, Figure 2I).

It has recently been shown that HSV-2 neutralization activity of sera collected from humans vaccinated with a gD subunit vaccine correlated with the presence of IgG Abs that target epitopes overlapping those of the gD-specific MAbs DL11, MC2, MC5, and MC23 (26). As sera derived from the guinea pigs immunized with gD + CpG exhibited a high HSV-2 neutralizing capacity, we examined if the Abs raised in guinea pigs after nasal immunization could target any of these known HSV-2 neutralizing epitopes. To address this, we tested the binding of purified guinea pig IgG Abs to the antigen in a competitive SPR assay by using six well-characterized gD-specific MAbs: 1D3, DL11, MC2, MC5, MC14, and MC23, all shown to exert HSV neutralizing properties singly or in combination with other MAbs (22, 27, 28). The MAbs 1D3, DL11, MC5, and MC14 bound efficiently to the gD protein immobilized on the sensor chip and thus included in the blocking SPR experiments. MC2 and MC23 MAb failed to bind efficiently to the gD protein on the sensor chip and were therefore omitted from further analysis (data not shown).

**Figure 3:**
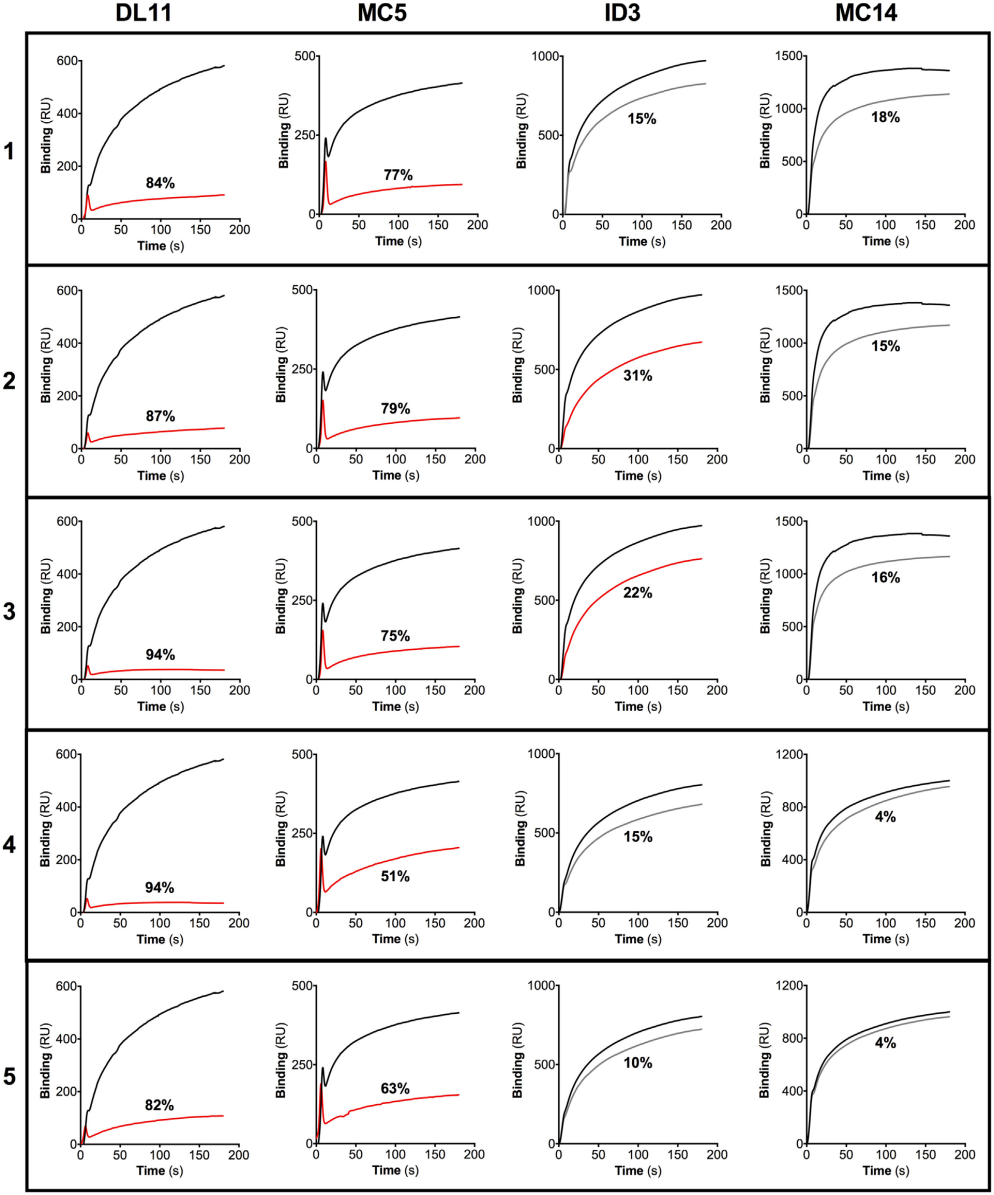
**IgG antibodies (Abs) raised after nasal immunization in guinea pigs targets epitopes overlapping those of known neutralizing gD monoclonal Abs (MAbs)**. IgG purified from sera of guinea pigs immunized nasally with gD + CpG were subjected to a biosensor surface plasmon resonance blocking assay using the gD-specific MAbs DL11, MC5, 1D3, and MC14. The binding between the individual MAbs and the gD protein immobilized onto the sensor chip was recorded by a biosensor, and the results are expressed as response unit (RU). A gD-coated sensor chip was then first exposed to purified guinea pig IgG after which the MAbs were injected sequentially after which the binding between the MAbs and gD was recorded. Data obtained from the association between the MAbs and the gD protein, in the absence of the blocking MAbs, are represented by black lines. Red lines show changes in RU of IgG samples with the ability to reduce the binding of the respective MAbs by more than 20%. Gray lines represent changes in RU of IgG samples with the ability to block the binding of the respective MAbs less than 20%. The percentage of reduction in the inhibition of the binding between the MAbs and the gD protein is shown for each IgG sample.

All purified IgG samples efficiently blocked the binding of the HSV-2 neutralizing MAbs DL11 and MC5 to the gD protein (51–94%). In addition, two of the guinea pig IgG Ab samples partially hindered the association of 1D3 to gD (22–31%), while none blocked the binding of MC14 more than 20% (Figure 3). These results indicate that nasal immunization with gD + CpG elicits a gD-specific IgG Ab response with high neutralizing capacity, targeting the two known neutralizing discontinuous epitopes recognized by DL11 and MC5 MAbs (Figure 3).

### Nasal Immunization Fails to Elicit Protective Immunity in Ab Deficient Mice

**Figure 4:**
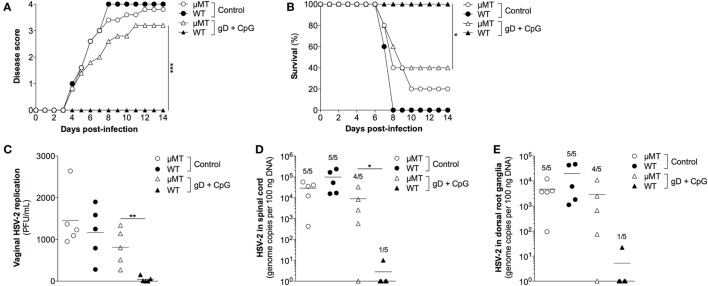
**The protective immunity conferred by nasal immunization is critically dependent on functional B cells**. Female μMT and wild-type C57BL/6 mice were immunized with gD together with CpG three times, with 1 week interval. Four weeks later, the immunized mice along with a PBS-treated control groups were given progesterone, followed 6 days later by a vaginal challenge with a lethal dose of HSV-2. **(A)** Disease progression after HSV-2 challenge (*n* = 5). Symptoms of the disease were scored as 0 = healthy, 1 = genital erythema, 2 = moderate genital inflammation, 3 = severe genital lesions, and 4 = hind limb paralysis for 14 days after challenge. **(B)** Survival rate after the viral challenge over a period of 14 days (*n* = 5). **(C)** HSV-2 levels in the vagina at day 3 after challenge, displayed as plaque forming units per mL (*n* = 5). **(D,E)** Spinal cord and DRG were collected at the time of sacrifice and tested for the presence of HSV-2 DNA (*n* = 5). Results displayed as genome copies per 100 ng sample DNA. The numbers shown above the bars represent the number of positive samples. Data points in **(C–E)** represent individual mice and the lines indicate the mean value of each group. Statistical significance for the end points in **(A)** and between the groups in **(C–E)** was calculated with a two-tailed unpaired *t* test and 95% CI. Survival **(B)** was analyzed by the Kaplan–Meier method and the Log-rank test. Differences were considered statistically significant at *p* values of <0.05 (*), <0.01 (**), and <0.001 (***).

Next, we evaluated the importance of gD-specific Ab responses induced by nasal immunization in protective immunity to genital herpes using mice lacking mature B cells (μMT mice). Groups of female age-matched C57BL/6 and μMT mice were immunized nasally with gD in combination with CpG ODN 1826 three times or were left unimmunized. Four weeks after the last immunization, mice were treated with progesterone followed by a vaginal challenge with a lethal dose of HSV-2. The gD + CpG immunized wild-type mice showed no symptoms of the disease (Figure 4A), 100% survival (Figure 4B) and no or very low viral loads in their vaginal secretions (Figure 4C).

Further, viral DNA was absent or very low in the spinal cord (Figure 4D) and the DRG (Figure 4E) of these mice compared with those of the control wild-type mice, indicating a neuroprotective effect of the immunization. In contrast, the nasally immunized μMT mice lacking mature B cells and Ab succumbed to genital HSV-2 infection (Figures 4A–C). Accordingly, high levels of viral DNA copies were detected in the spinal cord and the ganglia of the μMT mice (Figures 4D,E). These results indicate that Abs play an important role for protection against genital HSV-2 infection induced by nasal immunization.

## Discussion

We herein report that nasal immunization with CpG-adjuvanted HSV-2 gD protein confers protective immunity to primary genital herpes and recurrent outbreaks in guinea pigs. Furthermore, nasal immunization was shown to elicit high avidity, neutralizing IgG Abs directed against two discontinuous gD epitopes.

Earlier studies from our lab and others have documented the value of nasal immunization to mount protective immunity to primary genital herpes in mice (8–12). The potential of mucosal immunization with live attenuated/modified HSV-2 for prevention of latency in the guinea pig model of the disease has previously been reported (34–38). Furthermore, nasal immunization with recombinant HSV-2 gD together with the adjuvant LTK63 was shown to generate partial protection against primary genital herpes in guinea pigs (39). However, the impact of nasal immunization on induction of protection against genital herpes latency and recurrence in the clinically relevant guinea pig model of the disease remains obscure.

We found that nasal immunization with gD + CpG could induce significant protection to primary genital herpes infection as well as the establishment of latency and recurrent disease. The lumbosacral DRG of the immune guinea pigs, in sharp contrast to the controls, had no or very low levels of HSV-2 LAT. Ganglionic LAT transcript has been suggested to serve as the main surrogate of HSV reactivation and recurrent outbreaks (30, 33, 40). The level of LAT correlated with HSV-2 DNA found in the same DRG of the non-immune guinea pigs. This is in agreement with a study in which mutual presence of LAT and DNA was linked to the pattern of recurrent herpes (41).

We also showed that the establishment of latency and recurrences in guinea pigs is associated with the presence of IFN-γ mRNA in the DRG. This result is in line with previous reports indicating that IFN-γ production is associated with the establishment of latency in the trigeminal ganglia (HSV-1) and lumbosacral DRG (HSV-2) (32, 33, 42).

We documented that the nasal immunization of guinea pigs elicits systemic and vaginal specific IgG Ab responses. Furthermore, the sera from the nasally immunized guinea pigs had a potent HSV-2 neutralizing ability *in vitro*, and the neutralizing titers were found to correlate inversely with primary disease score, vaginal HSV-2 replication, and the number of recurrent days. The characteristics of a vaccine-induced Ab response remain poorly described in the HSV-2 guinea pig model. We demonstrated that the gD-specific serum IgG Abs raised following nasal immunization possess high avidity and target epitopes overlapping those of the neutralizing MAbs DL11 and MC5. Both of these MAbs have the ability to block downstream activation of the viral membrane fusion cascade. In addition, DL11 targets HSV binding to nectin-1 and HVEM and therefore neutralize HSV-2 efficiently (22, 43–47).

It has recently been shown in a recent large clinical trial of an exploratory vaccine composed of gD protein and AS04 adjuvant that neutralizing serum IgG Abs correlated with protection to genital HSV-1 infection (48). Importantly, the gD epitope profile of sera from our immunized guinea pigs matches that of vaccinated and naturally infected humans (26, 49), in that the sera of the immunized guinea pigs compete well with MAbs MC5 and DL11 but not with MAbs MC14 or 1D3. This is in contrast to sera from immunized rabbits, which contain Abs that readily block all four MAbs (unpublished data). The fact that humans and guinea pigs target the same gD epitopes lends further credence to the guinea pig model of genital HSV-2 infection.

None of the exploratory vaccines for genital herpes given by intramuscular injection has conferred protective immunity to genital HSV-2 in clinical settings [reviewed in Ref. (50)]. The mucosal route of immunization may thus be considered as an alternative route to mount protective immunity against genital herpes. Furthermore, the HSV gD protein has offered limited protection in human clinical trials. The value of inclusion of additional key HSV proteins in vaccine to enhance the breadth of vaccine-induced immunity has been underpinned by encouraging results from multivalent therapuetic HSV-2 vaccine candidates tested in phase II clinical trials (51, 52).

In summary, results presented herein provide new insights into the potential of nasal immunization for induction of protection against primary and recurrent genital herpes as well as the characteristics of the induced Ab response in guinea pigs. These results may inform a rational development of vaccines against genital herpes and presumably other sexually transmitted infections.

## Ethics Statement

This study was carried out in accordance with the recommendations of the Ethical Committee for Animal Experimentation in Gothenburg, Sweden. The protocol was approved by the Ethical Committee for Animal Experimentation in Gothenburg, Sweden.

## Author Contributions

Conceived and designed the experiments: JP, YZ, TO, FW, TC, and AH. Performed the experiments: JP, YZ, TO, KT, and FW. Analyzed the data: JP, YZ, TO, TC, FW, and AH. Wrote the paper: JP, YZ, TO, and AH. Edited the manuscript: JP, YZ, TO, TC, FW, QS, RE, GC, and AH.

## Conflict of Interest Statement

The authors declare that the research was conducted in the absence of any commercial or financial relationships that could be construed as a potential conflict of interest.
